# Supplementary material for: Early Prediction of Fetal Macrosomia Through Maternal Lipid Profiles
Source: Int J Mol Sci. 2025 Jan 28;26(3):1149. doi: 10.3390/ijms26031149 (PMC11818448; doi:10.3390/ijms26031149)
Supplement: Supplementary file 1 [file ijms-26-01149-s001.zip › ijms-3326272-Supplementary.pdf]

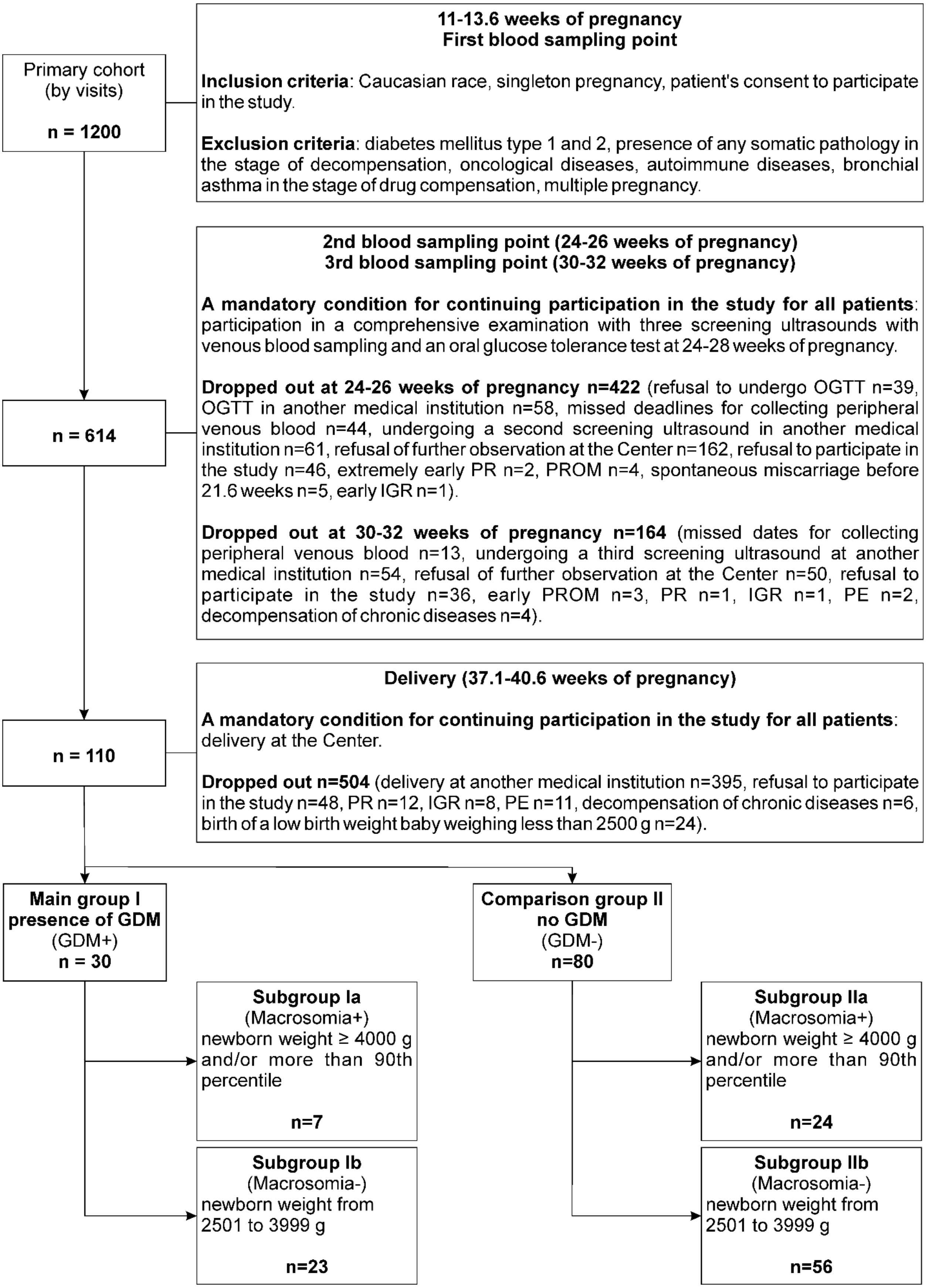

Figure S1. The design of the investigation.

Table S1. Clinical Characteristics of the Groups. Bold indicates *p*-values with statistical significance.

Parameter	Group		Value	*P*	Value	*P*
Age, years	GDM+* (n=30)	Macrosomia+* (n=7)	32 (29;35)**	0.56	32(29;36)	0.73
		Macrosomia-* (n=23)			32(28;34)	
	GDM-* (n=80)	Macrosomia+ (n=24)	31 (28; 34)		32(29;35)	
		Macrosomia- (n=56)			30(28;34)	
Weight before pregnancy, kg	GDM+ (n=30)	Macrosomia+ (n=7)	64 (55;77)	0.13	84(66;96)	**0.006**
		Macrosomia- (n=23)			63(55;67)	
	GDM- (n=80)	Macrosomia+ (n=24)	60 (55; 25)		64(58;68)	
		Macrosomia- (n=56)			59(54;63)	
Height, cm	GDM+ (n=30)	Macrosomia+ (n=7)	168 (164;170)	0.91	170(164;180)	0.33
		Macrosomia- (n=23)			168(164;170)	
	GDM- (n=80)	Macrosomia+ (n=24)	168 (164; 171)		169(168;170)	
		Macrosomia- (n=56)			167(164;171)	
Before pregnancy BMI, kg/m^2	GDM+ (n=30)	Macrosomia+ (n=7)	22.6 (20.1;26.5)	**0.03**	27(23;30)	**0.006**
		Macrosomia- (n=23)			22(20;24)	
	GDM- (n=80)	Macrosomia+ (n=24)	21.2 (19.5; 22.9)		22(20;26)	
		Macrosomia- (n=56)			21(19;22)	
Weight gain at the time of delivery, kg	GDM+ (n=30)	Macrosomia+ (n=7)	14 (11;17)	**0.009**	14(11;16)	**0.002**
		Macrosomia- (n=23)			11(9;14)	
	GDM- (n=80)	Macrosomia+ (n=24)	11(9;15)		16(13;18)	
		Macrosomia- (n=56)			13(11;16)	
Patient's newborn weight, kg	GDM+ (n=30)	Macrosomia+ (n=7)	3.5 (3.2;3.8)	0.86	3.6(3.5;4.1)	**0.004**
		Macrosomia- (n=23)	3.5 (3.2;3.7)		3.5(3.2;3.6)	
	GDM- (n=80)	Macrosomia+ (n=24)			3.8(3.5;4.1)	
		Macrosomia- (n=56)			3.4(3.1;3.6)	
Husband's newborn weight, kg	GDM+ (n=30)	Macrosomia+ (n=7)	3.6 (3.4;3.9)	0.78	3.6(3.5;3.8)	0.13
		Macrosomia- (n=23)			3.6(3.4;4)	

	GDM- (n=80)	Macrosomia+ (n=24)	3.6 (3.4;3.9)		3.8(3.6;4)	
		Macrosomia- (n=56)			3.5(3.3;3.8)	
The first-birth, number of patients (%)	GDM+ (n=30)	Macrosomia+ (n=7)	13 (43%)	0.67	2 (29%)	0.90
		Macrosomia- (n=23)			10 (43%)	
	GDM- (n=80)	Macrosomia+ (n=24)	30 (38%)		9 (38%)	
		Macrosomia- (n=56)			21 (38%)	
Gestational age at delivery, weeks	GDM+ (n=30)	Macrosomia+ (n=7)	39.0 (38.4;39.6)	0.17	40(39;40)	0.05
		Macrosomia- (n=23)			39(38;40)	
	GDM- (n=80)	Macrosomia+ (n=24)	39.5 (38.4;40.2)		40(39;40)	
		Macrosomia- (n=56)			39(38;40)	
Cesarean delivery, number of patients (%)	GDM+ (n=30)	Macrosomia+ (n=7)	18 (60%)	**0.02**	5 (71%)	**0.003**
		Macrosomia- (n=23)			13 (56%)	
	GDM- (n=80)	Macrosomia+ (n=24)	27 (34%)		14 (58%)	
		Macrosomia- (n=56)			13 (23%)	
Scheduled cesarean delivery, number of patients (%)	GDM+ (n=30)	Macrosomia+ (n=7)	13 (43%)	**0.01**	4 (57%)	**0.003**
		Macrosomia- (n=23)			9 (39%)	
	GDM- (n=80)	Macrosomia+ (n=24)	14 (18%)		6 (25%)	
		Macrosomia- (n=56)			8 (14%)	
Length of maternal hospital stay, days	GDM+ (n=30)	Macrosomia+ (n=7)	5(3;5)	**0.02**	5(3;5)	**0.007**
		Macrosomia- (n=23)			5(4;5)	
	GDM- (n=80)	Macrosomia+ (n=24)	4(3;5)		4(4;5)	
		Macrosomia- (n=56)			3(3;4)	
Newborn weight, grams	GDM+ (n=30)	Macrosomia+ (n=7)	3398 (3202;3873)	0.42	4200 (4082;4295)	**p< 0.001**
		Macrosomia- (n=23)			3250 (3150;3590)	
	GDM-	Macrosomia+ (n=24)	3495 (3207;4032)		4114 (4060;4244)	

	(n=80)	Macrosomia- (n=56)			3368 (3178;3538)	
Apgar score at 1 minute	GDM+ (n=30)	Macrosomia+ (n=7)	8(8;8)	0.53	8(8;8)	0.72
		Macrosomia- (n=23)			8(8;8)	
	GDM- (n=80)	Macrosomia+ (n=24)	8(8;8)		8(8;8)	
		Macrosomia- (n=56)			8(8;8)	
Apgar score at 5 minutes	GDM+ (n=30)	Macrosomia+ (n=7)	9(9;9)	0.74	9(9;9)	0.46
		Macrosomia- (n=23)			9(9;9)	
	GDM- (n=80)	Macrosomia+ (n=24)	9(9;9)		9(9;9)	
		Macrosomia- (n=56)			9(9;9)	
Length of newborn hospital stay, days	GDM+ (n=30)	Macrosomia+ (n=7)	4(3;5)	0.15	4(4;5)	**0.009**
		Macrosomia- (n=23)			3(3;5)	
	GDM- (n=80)	Macrosomia+ (n=24)	3(3;4)		4(3;5)	
		Macrosomia- (n=56)			3(3;4)	

* GDM+/ GDM- - the groups with/without gestational diabetes mellitus; Macrosomia+/ Macrosomia- - the subgroups with/without macrosomia.

** the data are presented as M(Q1;Q3), where M is the median, Q1 and Q3 are the first and third quantiles.
